# The effects of a plant-based and a plant- and marine-based n-3 oil supplement on behavioral reactivity, heart rate variability, and plasma fatty acid profile in young healthy horses

**DOI:** 10.1093/jas/skaf117

**Published:** 2025-04-08

**Authors:** Samantha Hartwig, Alexandra Rankovic, Persephone McCrae, Kiara Gagliardi, Scarlett Burron, Jennifer Ellis, David W L Ma, Anna K Shoveller

**Affiliations:** Department of Animal Biosciences, University of Guelph, Guelph, ON, CanadaN1G 2W1; Department of Animal Biosciences, University of Guelph, Guelph, ON, CanadaN1G 2W1; Department of Research and Development, Myant Inc., Toronto, ON, CanadaM9W 1B6; Department of Animal Biosciences, University of Guelph, Guelph, ON, CanadaN1G 2W1; Department of Animal Biosciences, University of Guelph, Guelph, ON, CanadaN1G 2W1; Department of Animal Biosciences, University of Guelph, Guelph, ON, CanadaN1G 2W1; Department of Human Health and Nutritional Sciences, University of Guelph, Guelph, ON, CanadaN1G 2W1; Department of Animal Biosciences, University of Guelph, Guelph, ON, CanadaN1G 2W1

**Keywords:** algal oil, behavior, camelina oil, equine nutrition, novel object test

## Abstract

Behavioral reactivity in horses poses a welfare and safety risk to both the horse and the handler, however, beneficial effects have been observed when dietary fat is increased in replacement of sugar. Supplementation with the fatty acids (**FA**) eicosapentaenoic (**EPA**) and docosahexaenoic acid (**DHA**) appear to improve negative behaviors in rodents and humans, but the effect of α-linolenic acid (**ALA**), EPA, and DHA, specifically, on reactivity in horses is unknown. The objective of this study was to evaluate the effects of camelina oil (**CAM**; ALA-enriched) and a mix of camelina and algal oil (**ALG**; ALA-, EPA-, and DHA-enriched) both fed at a dose of 0.37 g oil/kg body weight on plasma FA, behavior, and heart rate variability (**HRV**) in young horses compared to a negative control (**CON**). Thirty-four client-owned horses aged 7 mo to 6 yr were enrolled. Horses were assigned to either CAM, ALG, or CON and underwent a novel object test (**NOT**) before and after a 6-wk supplementation period. Prior to each NOT, blood was collected for evaluation of plasma FA profile (*n* = 28). During the NOT, behavior was recorded using a predetermined ethogram and assessed in BORIS software by 2 raters (*n* = 29). Electrocardiogram (**ECG**) data was collected at baseline, during the NOT, and after the NOT (recovery). The ECG data was analyzed in Kubios software for determination of heart rate (**HR**) and several HRV parameters (*n* = 24). The treatment oils were treated as fixed effects, baseline measurements as covariates, and location as a random effect. Plasma DHA (*P* < 0.01) was greater and n-6:n-3 ratio (*P* < 0.01) was reduced in ALG than in CAM and CON, while ALA and EPA were similar among treatments (*P* > 0.05). When treatments were pooled, the maximum HR (*P* < 0.01) and the low frequency to high frequency ratio HRV parameter (*P* < 0.01) were greater during the NOT compared to baseline and recovery. Bucking (*P* = 0.03) and backing (*P* = 0.02) behaviors were reduced in the CAM group compared to the CON group, but neither group differed from ALG. All other behaviors, HR, and HRV parameters were similar among treatments (*P* > 0.05). Our results suggest that the NOT was successful in creating acute stress, however, feeding either CAM or ALG at this dose did not reduce reactivity in this cohort of horses. Further research is needed to understand the effects of specific FA, if any, on behavior and HRV in more specific populations of horses and specifically those deemed highly reactive.

## Teaser

There is some evidence to suggest that behavioral reactivity in horses can be reduced by increasing fat in the diet. While plasma fatty acids were reflective of the oil consumed, supplementing horses with either camelina oil or a camelina and algae oil mix did not impact heart rate variability or most behavior parameters in horses as compared to a non-supplemented control.

## Introduction

Behavioral reactivity in horses can have consequences for the health and welfare of the horse, as well as the safety of the handler. Globally, the most common cause of injury related to equine activities is the result of a startled horse (Northey, 2003; Thomas et al., 2006; Guyton et al., 2013; Camargo et al., 2018; Acton et al., 2020). Nutritional interventions have been explored as a way to influence behavior in numerous species, including horses. Holland et al. (1996) observed that supplementing horses with soy lecithin and corn oil (10% of the diet) for 3 wk reduced spontaneous activity and tended to lower subjective reactivity scores across all fat supplemented diets. However, these results should be carefully interpreted as soy lecithin also contains phosphatidylcholine, which has been associated with improved cognition in Alzheimer’s disease and aging (Simpson et al., 2016; Kim et al., 2017; Li et al., 2019), suggesting a possible independent effect on behavior. Further, increased fat (9.9% to 11% of the diet) in replacement of sugar/starch fed for 2 to 9 mo reduced reactive responses to startle tests in foals (Nicol et al., 2005) and horses (Redondo et al., 2009), though the fat source was not disclosed. These results suggest that fat may influence equine behavior, but its effects independent of sugar/starch reduction and fat type require further study.

Associations between decreased circulating n-3 fatty acids (**FA**) and behavioral traits such as aggression have been identified in dogs and humans (Buydens-Branchey et al., 2003; Re et al., 2008). The long-chain n-3 FA eicosapentaenoic acid (**EPA**) and docosahexaenoic acid (**DHA**) may also influence neuroinflammation and the serotonergic system both in vitro and in vivo rodent and human models, suggesting a role in modulating mood and behavior (Schlicker et al., 1987; Speizer et al., 1991; Günther et al., 2010; Vedin et al., 2010; Patrick and Ames, 2015; Dang et al., 2018; Dong et al., 2018; Ali et al., 2020; Wang et al., 2021; Hu et al., 2022). Some studies suggest that long-chain n-3 supplementation may mitigate depressive-like behaviors, aggression, and behaviors associated with attention-deficit hypersensitivity disorder (ADHD) in rodents and humans (Buydens-Branchey et al., 2003; Richardson, 2014; Robertson et al., 2017). While direct links between n-3 FA and equine reactivity are limited, their role in aggression and mood modulation in other species suggests they may influence equine reactive behavior. However, despite some evidence that a higher fat diet may have a positive effect on behavior, influence of specific n-3 FA on reactivity-like behaviors in horses remains unknown. Nevertheless, n-3 FA appear to be involved in influencing certain behaviors in rodents and humans (Richardson, 2014; Dang et al., 2018; Wang et al., 2021; Hu et al., 2022), but further research is needed to understand the influence of specific n-3 FA on behavior in horses.

Camelina oil, derived from the oilseed crop *Camelina sativa*, is a plant-based source of the n-3 FA, α-linolenic acid (**ALA**; Burron et al., 2023; Richards et al., 2023). This is a novel supplemental oil used in equine diets to replace other common oils such as canola or flaxseed due to the greater shelf-life stability of camelina as compared to other oils (e.g., flaxseed oil), as well as the crop’s resistance to harsh climates and pests, making it a more sustainable option for providing n-3 FA to horses (Eidhin et al., 2003; Vollmann and Eynck, 2015; Ratusz et al., 2018). Once consumed, ALA can be converted via desaturases and elongases to the longer chain polyunsaturated EPA and DHA which can act as precursors to eicosanoids involved in the inflammatory response (Klenk et al., 1960 and Mead, 1968 as cited in Sprecher, 2000).

In human nutrition, fish oil is a common EPA and DHA source due to limited conversion of ALA to EPA/DHA (Emken et al., 1990). While the efficacy of conversion of ALA to EPA and DHA has not been explored yet in horses, incorporation of EPA and DHA into circulation and tissues has been observed following supplementation with ALA (Hansen et al., 2002; Hess et al., 2012), suggesting some conversion is occurring. To the authors’ best knowledge, no studies have been conducted that investigate the effects of ALA supplementation on behavioral reactivity in horses. Nevertheless, as the majority of literature focuses on the effects of EPA and DHA supplementation on behavior across other species, direct supplementation with long chain n-3 FA may be necessary to produce beneficial effects. However, as reviewed by Burron et al. (2024), fish oil is unsustainable due to dwindling fish stocks, and unable to meet the demands for human consumption, much less the demands for use in production and companion animal diets. The production of fish oil also raises various ethical and environmental concerns, such as unfavorable working conditions, overfishing, and habitat destruction (Auster et al., 1996; Jin et al., 2001; Bartek et al., 2021). Aside from this, fish oil often varies in quality, requiring extensive processing to remove contaminants and pollutants, such as polychlorinated biphenyls (Turon, 2013). Despite processing, however, some studies have revealed detectable, albeit mainly within acceptable, concentrations of various contaminants in fish oils (Fernandes et al., 2006; Rawn et al., 2009; Ozyurt et al., 2022). For these reasons, it is imperative to find an alternative source of EPA and DHA. Algal oil offers a more sustainable alternative with comparable EPA and DHA concentrations as fish oil. Algae can be cultivated in more controlled environments, eliminating the risk of contaminants and pollutants, and reducing the burden on marine ecosystems (Turon, 2013; Beal et al., 2018; Bartek et al., 2021). While fish oil has been well-accepted by horses in studies (O’Connor et al., 2004, 2007; Vineyard et al., 2010; Hess et al., 2012; Pearson et al., 2022), potential palatability concerns surrounding marine oils (i.e., fish or algae) may still deter owners. Since plant-based oils are readily accepted by horses (Holland et al., 1998; Delobel et al., 2008; Jerina et al., 2021) and contain the shorter-chain n-3 FA, ALA, the combination of a plant-based oil and marine-based oil may be a preferred approach.

Therefore, the objective of the current study was to evaluate the effects of feeding camelina oil or a camelina plus algal oil mixture for 6 wk on plasma FA profile, heart rate variability (**HRV**), and behavioral reactivity of young healthy horses as compared to a control group receiving no oil supplementation. We hypothesize that the plasma FA profile will reflect the oil consumed, the novel object test (**NOT**) will create stress as observed by changes to HRV, and that both camelina oil and the camelina plus algal oil mixture will reduce reactivity. Further, the camelina plus algal oil mixture will have a greater reduction in reactivity compared to camelina oil alone, due to the added benefits of EPA and DHA.

## Materials and Methods

### Animals

All experimental procedures were approved by the University of Guelph’s Animal Care Committee (Animal Use Protocol #5013) and were carried out in accordance with the national and institutional guidelines for the care and use of animals. Additionally, informed written consent was obtained from all horse owners, or authorizing agents, prior to the start of the study.

To be included in this study, horses had to be between the ages of 6 mo to 6 yr, deemed healthy based on physical examination by researchers and basic bloodwork (complete blood count and biochemistry), and could not have been on any supplemental oils for at least the past 6 mo. Horses were not screened for temperament by the researchers. Thirty-four client-owned horses (16 fillies, 6 colts, 9 geldings, 2 stallions, and 1 mare) with a mean age of 1.9 ± 1.7 yr (range: 7 mo to 6 yr), a mean estimated body weight (**BW**) of 399 ± 129 kg (range: 186 to 632 kg), and median body condition score (**BCS**) of 5 (range: 3.5 to 5.5) on the Henneke Scoring System (1 to 9; Henneke et al., 1983), were enrolled in this study. BW was estimated through calculation using measurements of heart girth and length (Carroll and Huntington, 1988).

Horses were recruited and enrolled from 7 different barns located within a radius of 180 km from Guelph, Ontario, Canada. While all barns differed in their management techniques, the management technique did not change for each barn throughout the 7-wk study. Management styles ranged from daily turnout (*n* = 19), housed outdoors only (*n* = 10), housed outdoors, but brought indoors during unfavorable weather (*n* = 4), and nightly turnout (*n* = 1). Most horses were maintained on a base diet of grain and either timothy or alfalfa/timothy hay (*n* = 28). However, 1 horse was maintained on just timothy/alfalfa hay (*n* = 1). Horses from 1 barn were maintained on grain and an unknown type of hay (*n* = 5) due to a timothy hay shortage from their supplier. Horse information, including distribution across barns, diet, and management can be found in the Supplementary Excel File S1. Horses enrolled in this study had no current illnesses or injuries, were not currently receiving any medications, and had not been supplemented with oil within the past 6 mo. A health check, including vitals and physical exam was conducted at the beginning of the study by the research team to ensure these criteria were met. Additionally, bloodwork collected from the baseline collections of NOTs was sent to the Animal Health Laboratory (AHL) at the University of Guelph (Guelph, Ontario) for serum biochemistry and complete blood count to ensure that there were no underlying abnormalities.

### Study design and experimental treatments

The NOTs and feeding trial took place from March 2024 to May 2024. Horses were balanced by location, age, and training level (in full training, started training/moderate training, or not yet started training). Horses were then randomly assigned 1 of 3 treatment oils: negative control (**CON**; *n* = 12), camelina oil (**CAM**; Smart Earth Camelina Corp., Saskatchewan; *n* = 11), or a camelina and algal oil (**ALG**; Veramaris, AX Delft, The Netherlands; *n* = 11) mix. Treatments were balanced as best as possible across barns. The FA composition of CAM and ALG can be found in Table 1. The control was made to look like oil by mixing xanthan gum (Duinkerken Foods Inc., Slemon Park, PE, Canada) with water at a ratio of 1:384, respectively. The ALG was created by combining 75% camelina oil and 25% algal oil by weight and gently inverting the bottle to mix.

**Table 1.** Fatty acid composition (% of total FA) of the camelina, algal oil, and camelina + algal oil mix fed to young horses at 0.37 g/kg BW for 6 wk

Fatty acid (% composition)	Camelina oil^1^	Algal oil^2^^,^^3^	Camelina + algal oil mix^1^^,^^2^
16:0	5.60	—	4.20
16:1n-7	0.00	—	0.00
18:0	2.60	—	1.95
18:1 *cis*-9	14.6	—	11.0
18:1n7	0.9	—	0.68
18:2n-6	19.3	—	14.5
18:3n-3	32.9	—	24.7
20:0	1.80	—	1.35
20:1n-9	13.9	—	10.4
20:1n-7	0.50	—	0.38
20:2n-6	1.80	—	1.35
20:3n-3	1.10	—	0.83
20:4n6	—	2.5	0.63
20:5n-3	—	14.5	3.63
22:1n-9	2.90	—	2.18
22:5n3	—	2.5	0.63
22:6n-3	—	45	11.3
Unsaturated fatty acids	89.0	—	66.8
Σ SFAs	10.5	27.5	14.8
Σ MUFAs	33.3	—	25.0
Σ PUFAs	55.7	70	59.3
Σ n-6	21.1	7	17.6
Σ n-3	34.4	66	42.3
n-6/n-3 ratio	0.61	0.11	0.42

Abbreviations: SFAs = saturated fatty acids; MUFAs = monounsaturated fatty acids; PUFAs = polyunsaturated fatty acids.

^1^Gas chromatography analysis by Smart Earth Camelina Corp. (Saskatoon, Saskatchewan, Canada).

^2^Veramaris Pets Algal Oil (Delft, Netherlands), mean values calculated using ranges given in technical data sheet.

^3^Algal oil was only fed when mixed with camelina oil, it was not fed as its own supplement.

Both the camelina and algal oils were tested prior to allocation into daily doses (approximately 2 to 3 wk prior to feeding) for primary oxidation compounds using a commercially available peroxide test kit (Peroxide Test Kit, MP Biomedicals, Irvine, CA, USA) following the manufacturer’s instructions. Peroxide value (**PV**) is the most widely used parameter to detect and quantify the degree and onset of the early stages of rancidity in oils and fats. Generally, PV should be below 10 to 20 mEq/kg fat to avoid rancid flavors (Kong and Singh, 2011). The PVs obtained were 1.44 and 0.74 mEq/kg for the camelina and algal oils, respectively, indicating that they were not rancid.

All horses underwent baseline blood collections and a NOT prior to starting the feeding trial. Horses were then supplemented with their assigned oil on top of their base diet for a period of 6 wk. The base diet for each horse was not changed. The feeding regimen began with a gradual acclimation period during the first week, starting with 0.05 g of oil/kg BW and increasing by 0.05 g of oil/kg BW increments each day to a final inclusion level of 0.37 g oil/kg BW by day 7. Daily oil doses were pre-measured by the research team and placed into light-protected amber-colored medicine bottles to prevent rancidity from light exposure (CP Lab Safety, California, USA). Owners were blinded to the oil that each horse was receiving. The bottles were labeled with the horse’s name, the day that they were to be given (e.g., day 1), and either “Oil A”, “Oil B”, or “Oil C” indicating CON, CAM, and ALG, respectively. Owners were instructed to store the oil in a cool and dark place (to prevent rancidity) and to top dress the entire contents of the bottle on their grain once daily. For the horse maintained on just hay, the oil was syringe fed once daily. One day after the final dose of oil was given, horses underwent a second round of blood collection, behavioral assessments, and HR measurements.

A total of 4 horses were removed from the study. Initially, 1 horse was withdrawn during baseline measurements as the horse was exhibiting signs of stress during handling, which prevented the collection of blood. Subsequently, 3 additional horses were excluded during the trial: 1 horse was relocated to a different barn, another was sold midway through and moved to a new barn, and a third developed lameness from a paddock injury near the end of the study, requiring anti-inflammatory medication. A total of *n* = 30 horses completed the trial (5 colts, 14 fillies, 8 geldings, 2 stallions, and 1 mare) and were an average age of 1.9 ± 1.7 yr (mean ± standard deviation) with an average BW of 408 ± 126 kg (mean ± standard deviation) and median BCS of 5 on a 9 point scale. The final treatment distribution was *n* = 8 in the CAM group, *n* = 10 in the ALG group, and *n* = 12 in the CON group.

### Fatty acid analysis

A small amount of EMLA cream (2.5% lidocaine and 2.5% prilocaine; Aspen Canada, Oakville, ON, Canada) was applied to the jugular groove approximately 30 min prior to blood collection to reduce discomfort from venipuncture. Approximately 20 mL of blood was collected from the jugular vein using a 20-guage multi-sample needle (Greiner Bio-One North America Inc, North Carolina, USA) into a serum vacutainer (Becton Dickinson, Franklin Lakes, NJ, USA) and K_2_EDTA vacutainer (Becton Dickinson) for the pre-study health check prior to supplementation, and a sodium heparin vacutainer (Becton Dickinson) for analysis of plasma FA profile prior to, and after the supplementation period. Vacutainers were inverted 8 to 10 times immediately after collection and placed on ice until centrifugation later that day. Samples were centrifuged for 15 min at 3,500 × *g* at 4 °C (Sorvall Legend RT Refrigerated Centrifuge, Thermo Fisher Scientific, Mississauga, ON, Canada). Plasma was then separated into aliquots and frozen at −80 °C until analysis.

For FA analysis, plasma samples were thawed on ice and once thawed, 50 µL of plasma was added to a 15 mL glass test tube (Thermo Fisher Scientific, Waltham, MA, USA) along with 950 µL of KCl (MilliporeSigma, Oakville, ON, Canada). Then, 4 mL of a chloroform methanol (CHCl_3_:MeOH) solution (2:1; MilliporeSigma) was added to each tube and the tubes were vortexed for 5 to 10 s (Benchmark BenchMixer Mandel, Guelph, ON, Canada). The contents of each tube were flushed with nitrogen for 5 to 10 s and then the tubes were capped tightly and were left to sit overnight (24 h) at 4 °C.

The next day, samples were centrifuged at 357 × *g* (RCF) for 10 min at room temperature (21 °C; Sorvall Legned RT+, Thermo Fisher Scientific, Mississauga) to separate the phases. The lower chloroform layer was then transferred into a new glass tube using a Pasteur pipette and left to completely dry down (approximately 30 to 45 min) under a gentle stream of nitrogen. Then, 2 mL of 0.2 KOH in methanol (Thermo Fisher Scientific, Waltham) was added to each tube, the tubes were capped and placed in an oven at 100 °C for 1 h to saponify. Once cooled, 2 mL of hexane (EMD Millipore, Oakville, ON, Canada) and 2 mL of 14% boron trifluoride-methanol solution BF_3_-MeOH (Sigma-Aldrich, Oakville, ON, Canada) were added to each tube. The tubes were capped again and placed in an oven at 100 °C for 1 h to methylate. Once cooled, 2 mL of double distilled water was added to each tube to stop methylation and the tubes were vortexed on the highest setting for 5 to 10 s (Benchmark BenchMixer Mandel). The tubes were spun down at 357 × *g* (RCF) for 10 min at room temperature (21 °C). The top hexane layer was then extracted and placed into a clean gas chromatography vial. The contents were completely dried down under nitrogen (approximately 30 to 45 min) and reconstituted in 100 µL of hexane (EMD Millipore) for gas chromatography analysis using the Agilent 6890 gas chromatograph (Agilent Technologies, Santa Clara, CA, USA). The FA peaks were identified and analyzed using the EZchrom Elite version 3.2.1 software (Agilent Technologies).

### Testing area

Each barn consisted of a slightly different testing area that was either an outdoor round pen, paddock, or an indoor arena that was split in half with jump poles with the effort to produce similar-sized testing areas. The average testing area size was 442 ± 193 m^2^ (114 to 743 m^2^). The same testing area was used for the baseline and final NOT for each horse. An iPhone 11 (Apple Canada Inc, Ontario, Canada) was mounted onto a moving tripod (PIVO Inc., California, USA) and placed outside of the testing area.

For each horse, 1 person was assigned to be the observer, and another person was assigned to be the record keeper for both the baseline and final NOT. The observer was responsible for the horse’s safety throughout the NOT, while the record keeper managed the video recording, stopwatch, and noted any interruptions or escapes. In some cases, an additional person was present to stabilize the tripod (e.g., during strong winds), and owners were sometimes allowed to be present to monitor their horses. For all NOT, the same team was present for each horse during both the baseline and final NOT, including the additional person and/or owners if present during the baseline NOT.

For 1 horse, the testing area was a small indoor arena (approximately 669 m^2^) that was not split in half at the owner’s request. There were no alternative areas outside of the arena for the tripod, observer, or record keeper to stand, so, they remained in a corner of the testing area throughout the test.

### Novel objects tests

**Figure 1.**
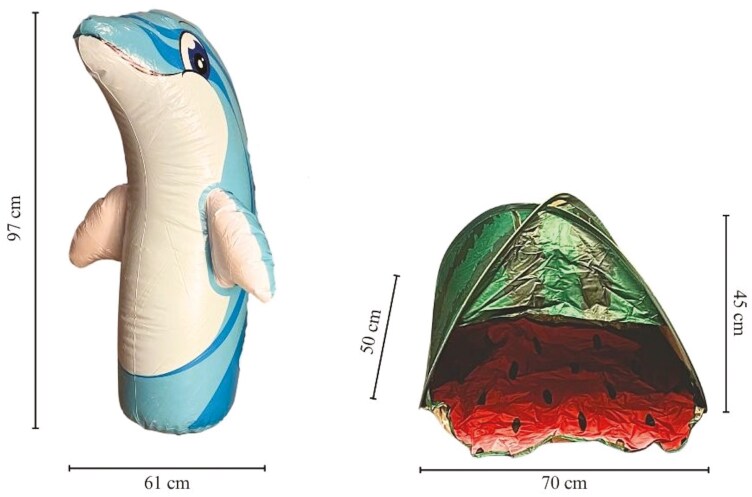
Images of the novel objects used during the baseline NOT (left) and the final NOT (right) for horses supplemented with 0.37 g/kg BW of either a placebo, camelina oil, or a camelina + algae oil mix for 6 wk, along with object dimensions. Note: images are not to scale.

A different novel object was used between baseline and final NOT to avoid habituation (Fig. 1). The first novel object used during the baseline NOTs was a medium-sized inflatable dolphin (Intex, California, USA) with a height of 97 cm and width (from fin to fin) of 61 cm when inflated. The second novel object used during the final NOTs was a medium-sized pet tent with a watermelon pattern (Amazon Canada, Ontario, Canada) with a height of 45 cm, a width of 70 cm, and a length of 50 cm. Both novel objects were chosen based on novelty to all horses enrolled as well as safety should the horse physically investigate the object during the test.

Prior to each NOT, horses were fitted with a smart textile girth band (Skiin Equine, Myant Inc., Ontario Canada) that had 5 embedded 4 cm square electrodes composed of silver- and carbon-coated yarns in a modified base-apex configuration. Each electrode was generously coated with salt- and chloride-free conductive gel (Spectra 360 Electrode Gel, Parker Laboratories Inc., New Jersey, USA), without shaving of the hair or other skin preparation. Electrocardiogram (**ECG**) data was recorded at a sampling frequency of 320 Hz and was transmitted via Bluetooth to a mobile phone (Motorola One Ace, Motorola Mobility LLC., Ontario, Canada). The smart textile girth band has been previously validated for the collection of HR and HRV data during rest and submaximal exercise in horses (McCrae et al., 2022, 2023). An elastic and silicone band was placed on top of the smart textile girth band to ensure good contact between the electrodes and skin. The horse remained unrestrained in a stall while a 30-min baseline ECG recording was obtained, with noise and interactions minimized throughout the 30-min duration. Once the baseline ECG recording was completed, a surcingle was fitted to the horse on top of the elastic wrap. A 90-min ECG recording was then started, and a phone was placed in a zippered pouch attached to the top of the surcingle to allow for continuous data collection during the NOT.

The horse was then led into the testing area by a familiar handler (either the barn owner or barn staff) and released by unclipping the lead rope from the halter. After releasing the horse, the handler exited the testing area but remained nearby to address any potential issues that could arise such as the horse escaping the testing area. The novel object was then placed in the center of the testing area by the research team and a timer for 15 min was set by the record keeper, indicating the start of the test. During this time, the horse was allowed to move freely around the testing area and explore the novel object. If the horse escaped the testing area, the observer or the record keeper would notify the familiar handler and the horse would be promptly returned using a lead rope to the testing area. The timer was not paused during escapes, but the time of escape and return was recorded for exclusion of behaviors and HRV measurements for later analysis. Once the 15 min was completed, the familiar handler re-entered the testing area to catch and lead the horse back into a stall. The surcingle was removed by the research team, but the smart textile girth band and elastic wrap remained on the horse. The horse remained in a stall for an additional 30-min to collect a posttest ECG recording, during which time all noise and interactions were minimized as with the baseline measurements. Once the 30 min were completed, the elastic wrap and smart textile girth band were removed from the horse and any remaining electrode gel was wiped off the hair with a washcloth.

For the first 2 horses that underwent the baseline NOT, the novel object was placed off to one side, near the perimeter of the testing area for ease of recording. However, as this wasn’t a feasible location in some of the testing areas, the placement of the novel object was switched to the center of the testing area for all remaining horses. The placement of the novel object was consistent between baseline and final NOT for each horse.

### HRV analysis

All raw ECG were manually reviewed and analyzed using Kubios software (Kubios HRV Scientific Version 4.1.0, Kuopio, Finland). A 15-min sample from the baseline ECG reading, the 15-min NOT, and the first 15-min recording of the recovery period when the horse was returned to a stall after the NOT (hereafter referred to as the recovery period) were marked within the ECG reading using the times recorded during testing days and were analyzed separately. The similar recording length between periods allowed for more accurate comparison (Eggensperger and Schwarzwald, 2017). Prior to calculation of the HRV parameters, ECG data were manually inspected to ensure accurate R peak detection of each heart beat (based on a typical PQRST wave). Any sections of noise (periods of interference or artifacts without R peaks) were manually identified and removed from analysis. Basic HR metrics were assessed, including maximum HR (**HR**_**max**_), minimum HR (**HR**_**min**_), and mean HR. Time-domain HRV parameters assessed included the root mean square of successive differences (**rMSSD**) to evaluate rapid changes in HRV and the standard deviation of N-N intervals (**SDNN**) to evaluate overall HRV (Shaffer and Ginsberg, 2017). The low frequency to high frequency ratio (**LF/HF**) was evaluated to assess sympathetic dominance (Shaffer and Ginsberg, 2017).

HRV data was collected during the baseline, NOT, and recovery period at both the baseline and final NOT on a total of 24 out of the 30 horses. Second degree atrioventricular blocks were observed in the baseline recordings of 3 of the 24 horses that HRV data was collected for. As this is common in resting horses (Raekallio, 1992), these horses were not excluded from analysis. The HRV data from 2 horses were excluded from analysis due to the poor quality of the ECG recording from the baseline NOT, resulting in limited useable data. Another horse’s data was excluded due to Bluetooth connectivity issues resulting in data loss. The HR and HRV data from the horse with unusable behavior data described previously was also excluded from HR analysis due to owner interference. Due to an error in the recorded start time for the recovery period during the second NOT for 1 horse, their data was also excluded. Lastly, during both the baseline and final NOT, 1 horse rolled during the NOT and the textile band lost connection with the mobile phone, resulting in a partial ECG recording which was excluded from analysis.

### Reactivity analysis

Behavioral coding was performed on all videos by 2 trained raters using the behavioral analysis software BORIS (BORIS V.7.13.6, Torino, Italy). All escapes or interruptions were removed from the behavioral analyses. A behavioral ethogram was developed by combining and adapting behaviors observed in previous studies utilizing NOT in horses (Table 2; Visser et al., 2001; Nicol et al., 2005b; McCall et al., 2006; Visser et al., 2008; Christensen et al., 2011, 2021; Bulens et al., 2015; Liehrmann et al., 2022). As the removal of interruptions, especially when a horse escaped from the testing area, resulted in some horses having less NOT time analyzed, frequency behaviors were expressed as frequency per minute, and duration behaviors were expressed as a % of total testing time. Each rater individually coded each horse’s NOT, and results were averaged between raters.

**Table 2.** List of behaviors evaluated in young horses during the baseline NOT and final NOT after supplementation with either 0.37 g/kg BW of either a placebo, camelina oil, or a camelina + algae oil mix for 6 wk

Behavior^1^	Description
Defecation—stationary	The horse defecates while standing still
Defecation—moving	The horse defecates while moving (e.g., while walking or trotting)
Calling	The horse makes a loud vocalization. A break in noise signifies the end of the behavior
Snorting	The horse forcefully expresses air from nostrils producing sound
Walking	The horse moves in a 4-beat gait
Trotting—normal	The horse moves in a 2-beat diagonal gait with relaxed tail and head/neck
Trotting—excited	The horse moves in a 2-beat diagonal gait with a raised tail and head
Other motion	The horse moves at a gait greater than trotting, including cantering or galloping
Head toss	The horse tosses his head up and down quickly. The horse may do this a couple times in 1 bout, but each toss of the head up should be counted as a separate behavior.
Buck	The horse quickly extends 1 or both of the hind legs in a “kicking” motion.
Rear	The horse raises both front legs from the ground and places all weight on the hind legs.
Flehmen response	The top lip of the horse curls up
Backing	The horse moves backward
Pawing	The horse extends 1 of the front legs in front of them on the ground in a sweeping motion. When both front hooves are flat on the ground will signify the end of this behavior.
Ears pinned	Ears parallel to the ground and facing backwards
Ears attentive	Ears facing forward or backwards and attentive/focused
Eye widening	Whites of the eye are visible, and eyes are attentive/focused
Heavy breathing	Horse is breathing heavily with big exhales and inhales. Breathing is audible.
Latency to approach	The time it takes for the horse to come within approximately 0.5 m of the novel object.
Physically Investigate	The time the horse spends sniffing, pawing, nudging, or physically interacting with the novel object
Focus	The time the horse spends focused on or looking in the direction of the novel object (includes time spent investigating the object)

^1^Ethogram was developed by combining and modifying behaviors previously used in NOTs in horses (Visser et al., 2001, 2008; Nicol et al., 2005; McCall et al., 2006; Christensen et al., 2011, 2021; Bulens et al., 2015; Liehrmann et al., 2022).

The intraclass correlation coefficient was calculated for each behavioral parameter to assess inter-rated reliability. The intraclass correlation coefficient (**ICC**), based on mean rating (*n* = 2), absolute agreement, and a 2-way mixed-effects model, was calculated by creating an ANOVA table in Microsoft Excel (v.16.16, Microsoft, Redmond, WA, USA) for each behavior and using the following the equation (Koo and Li, 2016):


$$\begin{array}{l} \frac{MSR-MSE}{MSR+\frac{MSC-MSE}{n}} \end{array}$$

Where MSR is the mean square of the rows, MSE is the mean square for error, MSC is the mean square for the column, and *n* is the number of subjects. The ICC values for each parameter were >0.95, indicating excellent inter-rater reliability.

Rear, Flehmen, and Ears Pinned behaviors were observed in fewer than 5 horses during each NOT and were therefore excluded from statistical analysis. Eye Widening and Heavy Breathing behaviors could not be accurately recorded due to issues related to weather conditions (when tests were conducted outdoors), lighting, camera angles, and distance, and were subsequently also excluded from statistical analysis. Additionally, Defecation—Moving and Trotting—Normal, which were observed in fewer than 5 horses at each test, were combined with Defecation—Stationary and Trotting—Excited, respectively, to form broader categories of Defecation and Trotting behaviors. At 2 of the barns, a barrier was placed in the arena to create the testing area, but this created the opportunity for escape during the test. As a large number of horses (*n* = 17) underwent the NOT in this type of testing area, an Attempted Escape behavior was added to the ethogram. This behavior included any attempt to escape the testing area causing movement to the barrier, including knocking down poles, as well as successful escape from the testing area.

Of the 30 horses that completed the trial, 29 horses completed both the baseline and final NOT and were included in the behavior analysis, with *n* = 8 horses in the CAM group, *n* = 9 in the ALG group, and *n* = 12 in the CON group. One horse’s data was excluded from behavioral analysis as the owner altered the testing area during the baseline NOT, significantly altering the horse’s behavior independent of the novel object.

### Statistical analysis

Statistical analyses of the data were performed using PROC GLIMMIX of the SAS Studio software (v.9.4, SAS Institute Inc., Cary, NC, USA). Horse was treated as the experimental unit and the treatment (oils) were treated as fixed effects. Baseline measurements were treated as a covariate and location was treated as a random effect and retained when significant. An ANOVA was performed to assess the effects of treatment on behavior, HRV, and plasma FA. Assumptions of normality and equal variance were evaluated using the Shapiro–Wilk normality test and by visually assessing residuals. A lognormal transformation was applied to behavior data with the exception of walking, other motion, and ears attentive, and to HR data with the exception of the LF/HF ratio. Additionally, a lognormal transformation was applied to the FA 15:0, 22:0, 24:0, 17:1c10, 22:1n9, 24:1n9, 20:5n3, and sum of polyunsaturated FA (**PUFA**). Least square (**LS**) means were used to assess differences in means of treatment. Significance was declared at *P*≤ 0.05 and trends were declared at 0.05 < *P*≤ 0.10. When fixed effects were significant, means were separated using Tukey–Kramer Adjustments. Results are presented as LS means with the 95% confidence intervals. The LS means and 95% confidence intervals for lognormal transformed data were presented as back-transformed values.

## Results

### Plasma FA

The data from the 4 horses that dropped out of the trial were not included in FA analysis. Additionally, blood samples of 2 horses (1 receiving CON, and the other receiving ALG) at week 6 were excluded from analysis due to human error, resulting in the inclusion of 28 out of the 30 horses in the FA analysis (*n* = 8 in the CAM group, *n* = 9 in the ALG group, and *n* = 11 in the CON group). Several FA in plasma were different among treatments at week 6 (*P* < 0.05; Table 3).

**Table 3.** Mean fatty acid % composition with 95% confidence intervals of saturated fatty acids (**SFA**), monounsaturated fatty acids (**MUFA**), and polyunsaturated fatty acids (**PUFA**) identified in plasma total lipids from horses (*n* = 28) fed 0.37 g/kg BW of either a control (**CON**), camelina oil (**CAM**), or a camelina and algae oil mix (**ALG**) for 6 wk

Fatty acid, %	Treatment			*P*-values	
	CON	CAM	ALG	Treatment	Covariate
14:0	0.39 (0.30 to 0.49)	0.31 (0.21 to 0.40)	0.31 (0.21 to 0.41)	0.04	0.20
15:0	0.22^ab^ (0.18 to 0.27)	0.20^b^ (0.16 to 0.25)	0.26^a^ (0.21 to to 0.32)	0.05	0.49
16:0	12.4^ab^ (11.4 to 13.5)	11.8^b^ (10.7 to 12.8)	13.0^a^ (11.9 to 14.1)	0.04	0.03
18:0	17.0 (16.5 to 17.6)	17.2 (16.5 to 17.9)	16.4 (15.7 to 17.1)	0.21	0.06
19:0	0.29 (0.24 to 0.33)	0.30 (0.25 to 0.36)	0.34 (0.29 to 0.40)	0.18	0.62
20:0	0.55 (0.49 to 0.60)	0.56 (0.49 to 0.62)	0.62 (0.56 to 0.68)	0.15	0.53
22:0	0.25^b^ (0.20 to 0.31)	0.29^ab^ (0.23 to 0.37)	0.31^a^ (0.24 to 0.38)	0.02	0.03
24:0	0.20 (0.17 to 0.23)	0.19 (0.16 to 0.22)	0.23 (0.19 to 0.27)	0.29	0.03
∑ SFA	31.3 (30.0 to 32.5)	30.9 (29.6 to 32.2)	31.3 (30.0 to 32.6)	0.75	0.64
16:1c9	0.70 (0.50 to 0.90)	0.56 (0.36 to 0.75)	0.57 (0.37 to 0.77)	0.06	0.01
17:1c10	0.25 (0.18 to 0.36)	0.22 (0.16 to 0.30)	0.25 (0.17 to 0.37)	0.70	0.35
18:1c9	8.33^a^ (7.77 to 8.89)	7.30^ab^ (6.63 to 7.97)	7.21^b^ (6.59 to 7.83)	0.02	0.02
18:1c11	0.95 (0.81 to 1.09)	1.06 (0.89 to 1.23)	1.22 (1.06 to 1.38)	0.06	0.34
20:1c11	0.41^b^ (0.14 to 0.69)	0.67^ab^ (0.39 to 0.95)	0.69^a^ (0.41 to 0.97)	0.02	0.59
22:1n9	0.61 (0.48 to 0.78)	0.56 (0.43 to 0.74)	0.80 (0.61 to 1.05)	0.08	0.50
24:1n9	0.43 (0.36 to 0.52)	0.48 (0.39 to 0.59)	0.57 (0.47 to 0.69)	0.13	0.20
∑ MUFA	11.6 (11.0 to 12.2)	10.8 (10.1 to 11.5)	11.2 (10.6 to 11.9)	0.23	<0.01
18:2n6	51.3^ab^ (49.9 to 52.7)	53.1^a^ (51.4 to 54.8)	49.5^b^ (47.9 to 51.1)	0.02	<0.01
18:3n3	3.12 (2.55 to 3.68)	2.62 (1.96 to 3.28)	2.79 (2.16 to 3.41)	0.48	0.01
20:2n6	0.45^b^ (0.40 to 0.50)	0.55^a^ (0.49 to 0.61)	0.47^ab^ (0.41 to 0.53)	0.05	0.03
20:3n6	0.39 (0.28 to 0.50)	0.38 (0.27 to 0.49)	0.42 (0.31 to 0.53)	0.55	0.07
20:4n6	1.40^b^ (1.24 to 1.55)	1.31^b^ (1.13 to 1.50)	1.72^a^ (1.55 to 1.90)	<0.01	<0.01
20:5n3	0.13 (0.02 to 0.67)	0.26 (0.08 to 0.85)	0.65 (0.21 to 1.98)	0.06	0.45
22:5n3	0.18^b^ (0.13 to 0.24)	0.20^b^ (0.14 to 0.27)	0.30^a^ (0.24 to 0.37)	0.01	<0.01
22:6n3	0.23^b^ (0.00 to 0.61)	0.28^b^ (0.00 to 0.70)	1.47^a^ (1.04 to 1.90)	<0.01	0.64
∑ PUFA	57.0 (55.2 to 58.9)	58.3 (56.2 to 60.5)	57.4 (55.3 to 59.5)	0.35	0.07
∑ n-6	53.5^ab^ (52.2 to 54.9)	55.3^a^ (53.7 to 57.0)	52.1^b^ (50.6 to 53.6)	0.02	0.01
∑ n-3	3.58^b^ (2.97 to 4.18)	3.22^b^ (2.51 to 3.93)	5.24^a^ (4.56 to 5.91)	<0.01	<0.01
∑ n-6/n-3	16.7^a^ (13.9 to 19.4)	19.6^a^ (16.4 to 22.9)	10.9^b^ (7.85 to 13.9)	<0.01	<0.01

Abbreviations: SFA = saturated fatty acids; MUFA = monounsaturated fatty acids; PUFA = polyunsaturated fatty acids.

^a,b^Values in a row with different superscripts differ significantly (*P* < 0.05).

Myristic acid (14:0) had a treatment effect (*P* = 0.04), however myristic acid only tended to be greater in ALG and CAM than CON (*P* = 0.08 and *P* = 0.07, respectively), but ALG and CAM did not differ (*P* = 1.00). The composition of both pentadecanoic (15:0) and palmitic (16:0) acids in plasma also had a treatment effect (*P* = 0.05 and *P* = 0.04, respectively). Pentadecanoic acid was greater in ALG than CAM (*P* = 0.04), but neither differed from CON (*P* = 0.19 and *P* = 0.56, respectively). Similarly, palmitic acid was greater in ALG than CAM (*P* = 0.03), but neither differed from CON (*P* = 0.38 and *P* = 0.29, respectively). Behenic acid (22:0; *P* = 0.03) was greater in ALG than CON (*P* = 0.02), but not different than CAM (*P* = 0.84). There was also a trend for behenic acid to be greater in CAM than CON (*P* = 0.08). All other saturated FA (**SFA**) did not differ among treatments (*P*≥ 0.15).

Oleic acid (18:1c9; *P* = 0.02) was greater in CON than ALG (*P* = 0.03) and tended to be greater than CAM (*P* = 0.06). Oleic acid was not different between ALG and CAM (*P* = 0.98). The opposite was seen for gondoic acid (20:1c11; *P* = 0.02) where it was greater in ALG than CON (*P* = 0.04) and tended to be greater than CAM (*P* = 0.06). Similar to oleic acid, gondoic acid was not different between ALG and CAM (*P* = 0.99). All other monounsaturated FA (**MUFA**) did not differ among treatments (*P*≥ 0.06).

Linoleic acid (**LA**; 18:2n6; *P* = 0.02) in plasma was greater in CAM than ALG (*P* = 0.01), but neither differed from CON (*P* = 0.25 and *P* = 0.22, respectively). Eicosadienoic acid (20:2n6; *P* = 0.05) was greater in composition in CAM than CON (*P* = 0.02), but neither differed from ALG (*P* = 0.15 and *P* = 0.54). Arachidonic (20:4n6; *P* < 0.01), docosapentaenoic (**DPA**; 22:5n3; *P* = 0.01), and DHA (22:6n3; *P* < 0.01) were all greater in ALG than in CAM (*P* < 0.01, *P* = 0.03, and *P* < 0.01, respectively) and CON (*P* < 0.01, *P* = 0.01, and *P* < 0.01, respectively), but CAM and CON did not differ (*P* = 0.69, *P* = 0.84, and *P* = 0.96). In plasma, EPA (20:5n3; *P* = 0.06) tended to be different among groups. All other PUFA did not differ among treatments (*P*≥ 0.35).

The sum of n-6 FA (*P* = 0.02) in plasma was greater in CAM than ALG (*P* = 0.02), but neither differed from CON (*P* = 0.22 and *P* = 0.32, respectively). The sum of n-3 FA (*P* < 0.01) was greater in ALG than CAM (*P* < 0.01) and CON (*P* < 0.01), but CAM and CON did not differ (*P* = 0.71). The opposite was seen for the n-6:n-3 ratio (*P* < 0.01) where the ratio was lower in ALG than CAM (*P* < 0.01) and CON (*P* = 0.01), but CAM and CON did not differ (*P* = 0.16).

### Heart rate variability

Results for HRV are reported in Tables 4 and 5. There were no treatment or treatment by time effects for any of the HRV parameters (*P*≥ 0.21). There were time effects for both HR_max_ (*P* < 0.01) and LF/HF (*P* < 0.01). The HR_max_ was greater in the NOT period than both the baseline period and the recovery period (*P* < 0.01 and *P* = 0.02, respectively), and the HR_max_ tended to be greater in the recovery period as compared to the baseline period (*P* = 0.10). The LF/HF ratio was also greater during the NOT period than the baseline and the recovery periods (*P* < 0.01 and *P* = 0.02, respectively), with no differences between the baseline and recovery periods (*P* = 0.56). The rMSSD also tended to differ among time periods (*P* = 0.06). All other HRV parameters did not differ among periods (*P*≥ 0.34).

**Table 4.** HRV parameters presented as least square means with 95% confidence intervals of 24 young horses during a 15-min baseline reading, the 15-min NOT, and a 15-min recovery period reading after being supplemented with either camelina oil (**CAM**), a camelina + algae oil mix (**ALG**), or a control (**CON**) for 6 wk

HRV metric	Trmt	Period			*P*-value		
		Baseline	NOT	Recovery	Period	Period × Trmt	Covar
HR_max_, bpm	CON	65.7 (51.5 to 83.8)	130 (99.3 to 170)	81.4 (63.7 to 104)			
	CAM	91.7 (69.3 to 121)	137 ± (100 to 186)	105 ± (77.2 to 142)	<0.01	0.69	0.06
	ALG	68.2 (53.6 to 86.9)	129 (99.4 to 167)	87.5 (68.1 to 112)			
	Mean^1^	74.3^b^ (62.5 to 88.4)	132^a^ (107 to 162)	90.7^b^ (77.5 to 104)			
HR_min_, bpm	CON	43.6 (40.2 to 47.3)	46.8 (40.5 to 54.2)	43.7 (39.3 to 48.5)			
	CAM	44.0 (39.9 to 48.4)	42.4 (35.2 to 51.1)	45.5 (40.0 to 51.7)	0.53	0.49	<0.01
	ALG	44.7 (41.2 to 48.4)	47.4 (41.1 to 54.8)	47.2 (42.5 to 52.5)			
	Mean	44.1 (41.7 to 46.6)	45.5 (41.2 to 50.2)	45.4 (42.5 to 48.5)			
HR_mean_, bpm	CON	55.2 (49.5 to 61.6)	61.8 (52.6 to 72.5)	57.2 (50.8 to 64.4)			
	CAM	59.9 (52.8 to 68.0)	58.4 (47.8 to 71.3)	62.1 (53.7 to 71.8)	0.34	0.51	<0.01
	ALG	56.4 (50.6 to 62.8)	63.5 (54.4 to 74.2)	61.0 (54.1 to 68.9)			
	Mean	57.1 (52.9 to 61.7)	61.2 (54.4 to 68.7)	60.1 (55.7 to 64.8)			
rMSSD, ms	CON	44.7 (34.6 to 57.7)	31.9 (20.1 to 50.7)	49.2 (38.0 to 63.9)			
	CAM	41.5 (30.9 to 55.8)	30.7 (17.5 to 53.9)	41.9 (30.5 to 57.6)	0.06	0.92	<0.01
	ALG	45.3 (35.8 to 57.4)	33.5 (21.2 to 53.0)	42.1 (32.5 to 54.6)			
	Mean	43.8 (37.8 to 51.6)	32.0 (23.9 to 42.8)	44.3 (37.7 to 52.1)			
SDNN, ms	CON	51.5 (41.0 to 64.7)	53.3 (39.7 to 71.5)	53.9 (43.9 to 66.1)			
	CAM	50.7 (38.6 to 66.5)	51.8 (36.1 to 74.3)	55.1 (42.8 to 70.8)	0.79	0.99	<0.01
	ALG	49.0 (39.3 to 61.2)	53.0 (39.5 to 71.1)	50.5 (41.2 to 62.0)			
	Mean	50.4 (43.6 to 58.2)	52.7 (43.8 to 63.4)	53.1 (46.7 to 60.4)			
LF/HF	CON	3.01 (2.30 to 3.72)	6.34 (4.09 to 8.59)	2.72 (1.31 to 4.12)			
	CAM	3.24 (2.40 to 4.08)	5.39 (2.60 to 8.17)	4.08 (2.35 to 5.80)	<0.01	0.70	<0.01
	ALG	2.69 (2.09 to 3.29)	6.18 (3.88 to 8.48)	3.67 (2.26 to 5.07)			
	Mean	2.98^b^ (2.48 to 3.48)	5.97^a^ (4.51 to 7.43)	3.49^b^ (2.61 to 4.37)			

^1^Mean value for the parameter at each period pooled across treatments.

Abbreviations: Covar, covariate; HR, heart rate; bpm, beats per minute; rMSSD, root mean square of successive beat-to-beat differences; ms, milliseconds, SDNN, standard deviation of beat-to-beat intervals; LF/HF, low frequency to high frequency ratio.

**Table 5.** HRV presented as least square means with 95% confidence intervals of 24 young horses fed either camelina oil (**CAM**), a camelina and algal oil mix (**ALG**), or a control (**CON**) for 6 wk pooled across the baseline, NOT, and recovery periods.

HRV Parameter	Treatment			*P*-value
	CON	CAM	ALG	
HR_max_, bpm	88.6 (75.5 to 104)	109 (90.1 to 133)	91.6 (78.1 to 107)	0.21
HR_min_, bpm	44.7 (40.7 to 49.1)	43.9 (39.1 to 49.3)	46.4 (42.2 to 51.0)	0.72
HR_mean_, bpm	58.0 (52.5 to 64.1)	60.1 (53.1 to 68.0)	60.2 (54.5 to 66.6)	0.84
rMSSD, ms	41.3 (32.2 to 52.9)	37.7 (27.8 to 51.0)	40.0 (51.2 to 52.9)	0.89
SDNN, ms	52.9 (43.9 to 63.8)	52.5 (41.8 to 66.0)	50.8 (42.2 to 61.3)	0.95
LF/HF	4.02 (3.19 to 4.86)	4.23 (3.21 to 5.25)	4.18 (3.34 to 5.02)	0.94

Abbreviations: HR, heart rate; bpm, beats per minute; rMSSD, root mean square of successive beat-to-beat differences; ms, milliseconds, SDNN, standard deviation of beat-to-beat intervals; LF/HF, low frequency to high frequency ratio.

### Behavior

Behaviors for the CAM, ALG, and CON group at week 6 can be found in Table 6. During the final NOT, after 6 wk of supplementation with either CAM, ALG, or CON, only the Buck and Backing behavior differed among treatment groups (*P* = 0.03 and 0.02, respectively). The frequency per minute of bucking was greater in CON than in CAM (*P* = 0.03), but neither differed from ALG (*P* = 0.78 and *P* = 0.13). The percentage of time spent backing was greater in CON than in CAM (*P* = 0.02), tended to be greater in ALG than CAM (*P* = 0.07) but CON did not differ from ALG (*P* = 0.99). Trotting also tended to be different among treatment groups (*P* = 0.09). All other behaviors did not differ among treatment groups (*P*≥ 0.13).

**Table 6.** Behaviors observed in horses (*n* = 29) during the final NOT as least square means with 95% confidence intervals after being supplemented with either a control (**CON**), camelina oil (**CAM**), or a camelina + algae oil mix (**ALG**) for 6 wk

Behavior	Treatment			*P*-value	
	CON	CAM	ALG	Trmt	Covar
Defecation^1^	0.11 (0.07 to 0.20)	0.08 (0.05 to 0.14)	0.13 (0.07 to 0.22)	0.39	0.15
Calling^1^	0.64 (0.37 to 1.08)	0.52 (0.27 to 1.01)	0.28 (0.14 to 0.58)	0.19	<0.01
Snorting^1^	0.22 (0.13 to 0.36)	0.15 (0.08 to 0.28)	0.10 (0.05 to 0.18)	0.13	<0.01
Walking^2^	25.8 (18.1 to 33.5)	28.1 (18.8 to 37.4)	26.7 (17.9 to 35.5)	0.93	0.02
Trotting^2^	15.8 (7.80 to 32.1)	5.59 (2.29 to 13.6)	12.5 (5.81 to 26.8)	0.10	<0.01
Other motion^2^	6.27 (2.16 to 10.4)	5.19 (0.51 to 9.88)	4.94 (0.57 to 9.31)	0.77	<0.01
Head toss^1^	0.32 (0.13 to 0.79)	0.32 (0.10 to 1.06)	0.15 (0.05 to 0.43)	0.42	0.09
Buck^1^	0.28^a^ (0.16 to 0.48)	0.08^b^ (0.04 to 0.16)	0.21^ab^ (0.10 to 0.43)	0.03	0.83
Backing^2^	0.50^a^ (0.25 to 0.99)	0.02^b^ (0.00 to 0.15)	0.46^ab^ (0.16 to 1.37)	0.02	0.03
Pawing^2^	1.49 (0.74 to 2.99)	0.83 (0.39 to 1.77)	1.73 (0.87 to 3.43)	0.29	0.03
Ears attentive^2^	60.7 (46.1 to 75.4)	63.6 (46.1 to 81.2)	60.1 (43.5 to 76.6)	0.93	0.05
Physically investigate^2^	0.98 (0.31 to 3.16)	1.80 (0.32 to 10.1)	1.46 (0.30 to 7.07)	0.81	0.93
Focus^2^	3.42 (1.47 to 7.93)	2.70 (1.38 to 9.89)	3.26 (1.16 to 9.15)	0.98	0.04
Escape attempt^3^	1.21 (0.43 to 3.46)	2.09 (0.80 to 5.46)	3.27 (0.39 to 27.6)	0.57	0.63
Latency to approach^4^	51.8 (16.7 to 161)	83.4 (12.5 to 577)	81.8 (18.5 to 361)	0.84	0.19

Abbreviation: Covar, covariate.

^1^Behavior measured as frequency of behavior over total minutes of test (freq/min).

^2^Behavior measured as percentage of total test spent performing the behavior (%).

^3^Behavior measured as number of times behavior was performed during test (freq).

^4^Behavior measured in seconds (s).

## Discussion

To the authors’ best knowledge, this is the first study to evaluate behavior in response to supplementation with an ALA-rich oil and an ALA + EPA/DHA-rich oil when compared to a negative control containing no dietary fat in young horses.

The majority of published studies across mammals investigating the incorporation of FA from dietary supplements into circulation or tissues focus on either an ALA-rich source or an EPA/DHA-rich source, rarely a combination of the 2. In the present study, a combination of the ALA-rich oil, camelina oil (approximately 35% ALA), and the EPA/DHA-rich oil, algae oil (approximately 15% EPA and approximately 45% DHA), was investigated and compared with solely camelina oil, and a negative control (no additional fat). Despite both CAM and ALG supplying high quantities of ALA, no difference was found in the plasma ALA composition among groups. Plasma FA are reported here as % composition, rather than absolute values, thus each FA % composition is not independent of each other. As ALA makes up < 5% of the composition of plasma, it is possible that the alteration of other FA contributed to the lack of difference in ALA composition among treatments. Additionally, the lack of difference among ALA in plasma despite CAM and ALG supplying high quantities of ALA may be attributed to differing basal diets, and thus differing basal FA intake, among horses.

Interestingly, the ALG group had a lower plasma LA composition (49.5%) than the CAM group (53.1%), but neither treatment group was different from the LA composition of plasma in horses receiving the control (51.3%). In agreement with the current study, horses supplemented with a marine-based oil (supplying approximately 0.01 to 0.03 g EPA/kg BW/d and approximately 0.02 to 0.05 g DHA/kg BW/d) had lower plasma LA % compositions than horses supplemented with ALA-rich flaxseed (Vineyard et al., 2010; Hess et al., 2012). However, unlike our study, LA % composition was also lower in horses supplemented with the marine-based oil than the control (non-supplemented) horses (Vineyard et al., 2010; Hess et al., 2012). Supplementation occurred for a longer duration (70 to 90 d) in both the studies by Hess et al. (2012) and Vineyard et al. (2010) compared to the supplementation duration of 6 wk (42 d) in the current study, suggesting that supplementation for more than 6 wk is warranted to reduce circulating LA. To support these hypotheses, Vineyard et al. (2010) measured plasma FA at 35 d of supplementation and observed that while the % composition of LA in plasma was reduced in the fish oil group compared to the flaxseed oil group, neither differed from the non-supplemented group, similar to the present study. Additionally, the similarity in plasma LA % composition in the ALG and CON group could be attributed to the LA supplied by the camelina oil (approximately 14.5%) in the ALG group, thus not resulting in as big of a reduction in plasma LA compared to supplementing with fish and/or algae oil alone as observed by Hess et al. (2012) and Vineyard et al. (2010). Similarly, the differences in arachidonic acid (**AA**; 20:4n6) among treatment groups can likely be attributed to dietary compositions of treatment oils, particularly the small amount of AA (0.63%) present in the ALG group.

As expected, horses in the ALG group had a greater plasma composition of DHA than CAM and CON. However, the composition of EPA was similar among treatment groups, though it did tend to be greater in the ALG group. Previous studies have observed increases in EPA and/or DHA in the plasma of horses supplemented with either a fish or algal supplement (O’Connor et al., 2007; Vineyard et al., 2010; Hess et al., 2012; Kouba et al., 2019). The ALG used in the study had a greater % composition of DHA (11.3%) than EPA (3.63%), thus providing a rationale for the greater difference in the plasma composition of DHA among groups than EPA. Another possible explanation for the lack of differences in EPA composition among treatments in the present study is that EPA was being converted to docosapentaenoic acid (DPA; 22:5n3). The % composition of DPA in the plasma of horses in the ALG group was greater than horses fed either CAM or CON. The combined impact of the conversion of EPA to DPA along with the small amount of DPA (0.63 %) provided by the algae oil could have contributed to this difference in plasma DPA composition.

In a previous study investigating the safety and efficacy of camelina oil as a supplement, following a similar design and dosing as the present study, plasma FA profile in response to camelina oil supplementation in horses was reported (Burron et al., 2023). The FA profile of horses in the CAM group in the current study closely resembles that observed in the study performed by Burron et al. (2023), except for the presence of EPA and DHA. Contrary to the Burron et al. (2023) study, where EPA and DHA were not detected in the plasma FA profile, both EPA and DHA were found in the plasma samples of all horses in the current study, even though neither the CAM nor the CON group received a dietary source of these FA to the authors’ best knowledge. Since samples were analyzed in different laboratories, these differences are likely attributable to differences in analytical techniques, which may affect the detection levels of various FA. Additionally, the presence of EPA and DHA in the plasma of horses in the present study, even those not consuming EPA and DHA (i.e. CAM and CON groups) to the authors’ knowledge, suggests that horses have the capacity to convert some ALA to EPA and DHA.

The diet of each horse enrolled (excluding horses that dropped out mid-trial) consisted of both a concentrate/grain and a forage, however, the concentrate and forage type differed among horses (see Supplemental Materials S1). Additionally, as all horses either had daily turnout or were housed outdoors with no turn-in, it is possible that horses had access to pasture, though the time of year that the study was conducted (early March–Early May) likely limited pasture availability. Thus, since fresh pasture grasses contain greater concentrations of ALA than hay (Glasser et al., 2013), if some horses were consuming more pasture than others, basal ALA intake would also differ. Similarly, the FA profile varies depending on the concentrate and forage type, and within the same forage type, factors such as location or supplier can further alter the FA profile (Glassier et al., 2013; Burron et al., 2024). Overall, differences in diet composition combined with variations in concentrate and forage intake likely resulted in differing basal FA intake among horses. Therefore, while some of the differences in plasma FA profile observed in the present study may be attributed to the addition of the treatment oils (i.e., CAM or ALG), as horses were consuming differing basal diets, it is possible that differences in basal intake of FA may also be contributing to these differences.

During periods of stress, HR will increase while HRV parameters, such as rMSSD and SDNN, will decrease in mammals (Shaffer and Ginsberg, 2017). Further, an increase in the LF/HF ratio indicates a shift from parasympathetic to sympathetic function, which also occurs during stress (Shaffer and Ginsberg, 2017). These changes to HR and HRV have been observed in horses in situations that are considered stressful such as transport, handling tests, social isolation, and exercise (Visser et al., 2002; Rietmann et al., 2004; Schmidt et al., 2010a, 2010b; Younes et al., 2016; Reid et al., 2017; Ohmura and Hiraga, 2022). Thus, the changes observed in the HR and HRV of horses in the current study across the NOT periods (baseline, NOT, recovery) indicate that the NOT successfully induced stress.

Unexpectedly, the treatments in the current study did not affect HR or HRV parameters. Serum concentrations of EPA, DPA, and DHA were observed to be associated with lower resting HR, with EPA having a greater association than DPA and DHA in men (Tajik et al., 2018). Further, the literature is fairly consistent regarding the effects of fish oil (EPA and DHA) on HR in humans, whereby HR is reduced during both rest and exercise when supplemented fish oil (providing 0.005 to 0.011 g EPA/kg BW/d and 0.008 to 0.022 g DHA/kg BW) compared to a non-supplemented group (Geelen et al., 2005; Ninio et al., 2008; Peoples et al., 2008; Buckley et al., 2009). Horses in the present study were receiving greater amounts of EPA and DHA per day (approximately 0.01 g EPA/kg BW and approximately 0.04 g DHA/kg BW, based on calculated values in Table 1) than the above studies, thus dose is unlikely to be contributing to the lack of effects on HR. Additionally, in a meta-analysis, Mozaffarian et al. (2005) observed little evidence for a dose-response effect for fish oil (dose of EPA + DHA ranging from approximately 0.01 to 0.20 g/kg BW/d) supplementation on HR in humans. In horses supplemented with 0.324 g of fish oil per kg BW, HR during exercise was lower than in horses supplemented with the same dose of corn oil (O’Connor et al., 2004). The differences in the amount of EPA and DHA, and the EPA:DHA ratio among various species of fish oil and between fish and algal oil should be taken into consideration when comparing results among studies. Hence, the reduced EPA compared to DHA (EPA:DHA ratio of approximately 1:3) provided to horses in the current study as compared to the horses in the O’Connor et al. (2004) study (EPA:DHA ratio of approximately 1:1) could be contributing to the differing results. Aside from dose, the absence of an n-6 enriched oil group in the current study could explain why our results differ from those of O’Connor et al. (2004). While studies in humans typically compare fish oil to a placebo, much like our study, the base diet of humans typically contains greater n-6 FA than the base diet of a horse. Thus, it is still possible the lack of treatment differences in the present study could be at least partially due to the absence of an n-6 enriched oil group. Reduction in HR following supplementation with a source of EPA/DHA (e.g., fish oil) is suggested to be due to the ability of EPA and DHA to increase the strength of stimulus needed to create an action potential in cardiac myocytes (Kang et al., 1995). While this mechanism of action may occur in both physical (e.g., exercise) and psychological (e.g., exposure to a novel object) stress, exercise may cause a greater increase in HR than psychological stress. Further, HR has been observed to be greater in horses and ponies fed a high-energy diet resulting in a greater BCS than in horses and those fed restricted energy or control diet (D’Fonseca et al., 2021; Jansson et al., 2021). It is possible that the effects of the fish oil are more prominent when HR is greater, thus the type of stress, the health status of the horse, and resulting HR increase may influence the results. Additionally, carbohydrates are the primary substrate utilized for energy in short-term exercise (reviewed by Lawrence, 1990), whereas acute psychological stress (i.e., exposure to a novel object) may result in greater fat utilization for energy due to release of glucocorticoids (reviewed by MacFarlane et al., 2008). Therefore, in cases of acute psychological stress, fats may be more directed towards meeting the energy demands of the stress response rather than contributing to other physiological roles of n-3 FA, such as modulating neuro-inflammation and altering stress-related behaviors.

The lack of differences among treatments for HRV parameters was also unexpected. In humans, consumption of fish has been positively correlated with the improvement of HRV parameters (Christensen et al., 2001; Mozaffarian et al., 2008). Further, fish oil supplementation (approximately 0.01 to 0.04 g EPA/kg BW/d and approximately 0.01 to 0.04 g DHA/kg BW/d) appears to increase the HRV parameters SDNN and rMSSD in healthy and diseased states (Christensen et al., 1996, 1999). While a dose response (approximately 0.001 to 0.04 g EPA/kg BW/d and approximately 0.01 to 0.04 g/kg BW/d) may exist for HRV in humans (Christensen et al., 1999; Sjoberg et al., 2010), factors aside from dose may be influencing the results of the present study. In overweight adults at rest, HF power was greater (indicating enhanced parasympathetic function) and the LF/HF ratio of overweight adults tended to decrease over time when supplemented with fish oil, providing 0.36 g EPA and 1.56 g DHA per day (approximately 0.005 g EPA/kg BW/d and 0.02 g DHA/kg BW/d), for 12 wk as compared to a sunflower oil supplemented group (Ninio et al., 2008). Conversely, miniature pigs supplemented with fish oil (containing 7.5% EPA and 23.8% DHA and making up 12% of the diet as fed) for 10 wk had a greater LF/HF ratio at rest than those fed either lard (high in saturated fats) or sunflower oil (Malbert et al., 2022). While the doses of fish oil provided by Ninio et al. (2008) and Malbert et al. (2022) differ, the opposing results may also indicate species-specific differences in response. To the authors’ best knowledge, this is the first study to investigate HRV response to EPA and DHA supplementation in horses, thus it is possible that the results observed in the current study could be attributed to species-specific differences in the HRV response to EPA and DHA.

There is considerably less literature surrounding ALA supplementation on HR and HRV. Previous literature in humans and horses suggests that ALA supplementation does not affect HR, despite ALA having a similar impact as EPA and DHA on cardiac myocyte action potentials (Kang et al., 1995), but may affect certain HRV parameters (Christensen et al., 2005; Ebbesson et al., 2010; Mowry et al., 2022; Lyudinina et al., 2024). Similar to the present study (providing 0.09 to 0.12 g ALA/kg BW/d), the resting HR of horses supplemented with flaxseed oil (supplying approximately 0.11 g ALA/kg BW/d) was not different from the HR of horses fed either rice bran oil (supplying approximately 0.06 g ALA/kg BW/d) or no oil supplementation (Mowry et al., 2022). Similarly, 2 separate studies in human populations, 1 in Indigenous peoples of Alaska between the ages of 35 and 74 yr that varied in health status and the other in highly trained male cross-country skiers aged 18 to 29 yr, observed no association between plasma ALA and HR at rest (Ebbesson et al., 2010; Lyudinina et al., 2024). Supplementation with ALA, however, does appear to be associated with positive changes in some HRV parameters in humans in healthy and diseased states (Christensen et al., 2005; Lyudinina et al., 2024). The results from the current study only partially agree with previous findings. No differences in HR (max, min, mean) or HRV (rMSSD, SDNN, LF/HF) were observed among treatments, regardless of ALA compositions. It is possible that the lack of treatment differences for HRV could be due to the lack of differences in plasma ALA of horses in the present study. However, given that plasma EPA was elevated in horses in the ALG group with no associated changes in HR or HRV, it is more likely that the lack of treatment differences is due to other factors such as individual variability among horses (e.g., age) or variability in basal diet.

Age may also influence HR and HRV responses in horses, for example, younger horses (aged 4 to 6 yr) were observed to have greater HR, but lower HRV measurements than aged horses (Younes et al., 2016; Janczarek et al., 2020). Further, in Thoroughbred horses at different ages (24-h to 30-yr old) and stages of training, Ohmura and Jones (2017) described the relationship between age and HRV to be hyperbolic. Horses in the current study varied in age, ranging from 7 mo to 6 yr. While treatments were balanced across age, the range in age of horses included in this study could have increased overall variability and made it difficult to detect differences among treatments.

While oil supplementation did not have any adverse effects on behavior in the present study, the lack of treatment differences in most of the behaviors assessed was unexpected. Both the frequency of bucking and duration of time spent backing up were reduced in horses fed CAM compared to CON, however, both behaviors constituted a minimal part (0.08 to 0.28 bucks/min and 0.02% to 0.50% of time spent backing) of the horse’s time budget during the NOT. In contrast, reduced reactivity during startle tests has been observed when horses are fed diets high in fat and low in starch/sugar as compared to diets low in fat and high in starch/sugar (Holland et al., 1996; Nicol et al., 2005; Redondo et al., 2009). The startle tests differed among studies, with Holland et al. (1996) using the opening of an umbrella to elicit a response, Nicol et al. (2005) using both human and NOTs in addition to a handling test across a novel bridge or tarp, and Redondo et al. (2009) using a moving object in their startle test. While our HR and HRV data provided evidence to suggest that acute stress was achieved during our NOT, differing protocols to elicit acute stress may influence the behavioral response to oil supplementation. Additionally, it is possible that the dose of fat or the length of supplementation may play a role in altering behavior. Horses were supplied with approximately 2 to 2.5 g of fat/kg BW in the above studies, whereas horses in the present study were only receiving 0.37 g of fat/kg BW. While the studies by Nicol et al. (2005) and Redondo et al. (2009) had longer supplementation periods than the current study (8 and 34 wk, respectively), Holland et al. (1996) observed differences in behavior after 3 wk of fat supplementation, suggesting the lack of behavioral response in the current study may be due to other factors such as variability among horses and basal diets, specifically variability in starch/sugar content of basal diets. However, given the minimal behavioral impacts observed in the present study when starch/sugar in the base diet was not altered over the course of the study, it is possible that the behavioral impacts in horses observed in previous literature could be attributed more to the reduced starch/sugar content of the diets and that oil treatment may not impact reactivity as previously hypothesized. Thus, it is hard to determine whether the addition of fat reduced reactivity, or whether increased starch/sugar content worsens behavior. Elegant experimental designs need to be implemented to tease these dietary factors apart.

Various studies in rodents, cats, and humans have investigated the effects of EPA and DHA on sickness-induced behaviors, likely resulting in low n-3 status, in comparison to a non-supplemented control or n-6 supplemented group (Corbee et al., 2013; Richardson, 2014; Dang et al., 2018; Wang et al., 2021; Hu et al., 2022). While research pertaining to fear- or reactivity-like behaviors is limited in the horse and other prey species, the effects of EPA and DHA on sickness-induced behaviors suggest a potential modulating role of these FA in other types of behaviors as well. It is possible that behavioral modifications due to dietary fat may be more pronounced in populations experiencing behavioral concerns, or in certain mental disorders that may alter behavior such as anxiety- or depression-related behaviors. While no health concerns were reported in the study by Nicol et al. (2005), foals were in the process of weaning which can cause stress and associated negative behaviors (Erber et al., 2012; Delank et al., 2023). Similarly, high starch/sugar content of diet can cause increased incidence of gastric ulcers and GI concerns (Luthersson et al., 2009; Andrews et al., 2017). Therefore, the lack of obvious illness or injury in horses in the present study could be a contributing factor to the lack of treatment differences in behavioral parameters. Further, even though we successfully induced acute stress during the NOT, unlike some of the behavioral studies discussed, horses were not experiencing any chronic stress, such as weaning. Therefore, improvements in behavioral reactivity in response to fat supplementation may be more pronounced in horses experiencing physical (i.e., injury or illness) or prolonged psychological stress.

A notable limitation of this study is the inherent variability among horses. Although extensive efforts were made to locate a single barn with a sufficient number of horses to constitute the entire study population, this goal could not be achieved. Consequently, horses were sourced from multiple locations, introducing variability that may have impacted the study’s outcomes. Additionally, the age range of horses enrolled was increased to allow the fulfillment of the desired sample size. To reduce the impacts of this variability, however, treatment groups were balanced by age, location, and sex as best as possible. Further, during statistical analysis, location was used as a random variable where possible (as its inclusion would occasionally over-parametrize the model) and baseline measurements were used as covariates to account for individual variability. Several parameters showed significance for the baseline covariate, indicating that baseline values have an impact on the final results. Nevertheless, while variability was carefully accounted for in both study design and statistical analysis, it is possible that overall variability in the study population could have made it difficult to detect differences in behavior and HRV among treatments. Another major limitation would be the differences in basal diets between horses. Even among horses consuming the same type of forage (e.g., timothy/alfalfa hay), FA content of the forage consumed may differ depending on location or supplier and overall intake may vary. While the only dietary change that occurred during the trial was the addition of a treatment oil or a control, differing initial fat and FA intake could have impacted the results. Future studies should investigate the effects of oil supplementation on behavioral reactivity and HRV in more specific populations consuming the same basal diet to reduce variability. Moreover, as discussed above, behavioral alterations in response to fat supplementation appear to be more pronounced in susceptible populations, such as horses undergoing a stressful life stage (e.g., weaning) or experiencing an illness or injury that may negatively impact behavior. Thus, the effects of ALA and an ALA + EPA/DHA supplement on behavioral reactivity should be investigated in susceptible populations of horses.

## Conclusions

In summary, supplementing young, healthy, and lean horses with camelina oil (source of ALA) or a camelina and algae oil mix (source of ALA and EPA/DHA) at a dose of 0.37 g oil/kg BW, resulted in plasma FA compositions that were reflective of the oil consumed. While some differences in behavior parameters were observed, supplementation with camelina or the camelina and algal oil mix overall had minimal effects on behavior when considering all of the parameters assessed and did not significantly impact HR or HRV during the NOT. Thus, supplementation with camelina oil or a camelina and algae oil mix may not be an effective method for reducing behavioral reactivity in healthy horses that are not experiencing any chronic stress. More research is required to understand the impacts of specific FA, if any, on behavioral reactivity and HRV.

## Supplementary Material

skaf117_suppl_Supplementary_Material

## Abbreviations

AHL: Animal Health Laboratory
ALA: α-linolenic acid
BCS: body condition score
BW: body weight
DHA: docosahexaenoic acid
DPA: docosapentaenoic acid
ECG: electrocardiogram
EPA: eicosapentaenoic acid
FA: fatty acid
HR: heart rate
HRV: heart rate variability
ICC: intraclass correlation coefficient
LA: linoleic acid
LF/HF: low frequency to high frequency ratio
MSC: mean square of columns
MSE: mean square of error
MSR: mean square of rows
MUFA: monounsaturated fatty acid
NOT: novel object test
PUFA: polyunsaturated fatty acid
rMSSD: root mean square of successive differences
SDNN: standard deviation of N-N intervals
SFA: saturated fatty acids
